# The Mediating Role of Social Support Between Pregnancy Anxiety and Emotional Suppression in Women with Threatened Preterm Labor

**DOI:** 10.3390/jcm14176002

**Published:** 2025-08-25

**Authors:** Joanna Grzesik-Gąsior, Katarzyna Zalewska, Agnieszka Pieczykolan, Sebastian Kowalski, Karolina Żak-Kowalska, Iwona Niewiadomska, Agnieszka Bień

**Affiliations:** 1 State University of Applied Sciences in Krosno, 38-400 Krosno, Poland; katarzyna.zalewska@pans.krosno.pl (K.Z.); sebastian.kowalski@pans.krosno.pl (S.K.); 2 Chair of Obstetrics Development, Faculty of Health Sciences, Medical University of Lublin, 20-081 Lublin, Poland; agnieszka.pieczykolan@umlub.edu.pl (A.P.); agnieszka.bien@umlub.edu.pl (A.B.); 3 New Medical Techniques Specialist Hospital of the Holy Family, 36-060 Rudna Mała, Poland; zak.karolinajagoda@icloud.com; 4 Department of Social Psychoprevention, John Paul II Catholic University of Lublin, 20-950 Lublin, Poland; iwona.niewiadomska@kul.pl

**Keywords:** pregnancy, social support, pregnancy anxiety, emotions regulation, premature birth

## Abstract

**Background**: Threatened preterm labor is associated with heightened emotional distress in pregnant women, including anxiety, guilt, and depressive symptoms. Effective coping relies on psychosocial resilience, particularly emotional suppression and perceived social support. This study examined the mediating role of social support in the relationship between anxiety and emotional suppression. **Methods**: The study was conducted in Poland between December 2024 and June 2025 among 213 women hospitalized due to threatened preterm labor. Participants completed the Berlin Social Support Scales, the State–Trait Anxiety Inventory, the Courtauld Emotional Control Scale, and a structured interview. **Results**: Women hospitalized for threatened preterm labor have moderate levels of anxiety as a state (M = 44.08 ± 10.59) and trait (M = 39.75 ± 9.99). Mediation analyses revealed that two dimensions of social support—perceived available support and Buffering–Protective support—significantly influenced the relationship between anxiety and emotional suppression (anger, depression and anxiety). In contrast, need for support, support seeking, and Currently Received Support were not significant mediators. **Conclusions**: The results indicate a complex interplay between anxiety, perceived support, and emotional suppression. The hypothesized simple buffering model was not confirmed. However, perceived available support was associated with reduced emotional suppression, suggesting a protective role. Buffering–Protective Support showed an activating effect, possibly encouraging emotional inhibition in stressful situations. These results underscore the importance of assessing perceived social support in clinical settings and tailoring psychological interventions for pregnant women at risk of preterm labor.

## 1. Introduction

The emotional state of a pregnant woman is a significant predictor of pregnancy progression and birth outcomes. Pregnancy-related anxiety may arise from uncertainty regarding labor and concerns about the newborn’s health, particularly among women with high-risk pregnancies [1]. The likelihood of experiencing anxiety during pregnancy increases in the context of adverse socioeconomic conditions, a complicated obstetric history (e.g., miscarriage or stillbirth), and insufficient or inconsistent social support. In extreme cases, this anxiety can manifest as a specific anxiety disorder known as tokophobia [2,3]. Emotional suppression plays a crucial role in mitigating the negative effects of anxiety.

During pregnancy, a commonly used emotion regulation strategy is the suppression of negative affect, defined as the deliberate inhibition of emotions such as anger, anxiety, or depression. This strategy may temporarily reduce emotional expression and foster a sense of psychological control. However, chronic emotional suppression has been associated with adverse mental and physical health outcomes. Higher levels of emotional suppression do not reflect adaptive regulation but rather indicate a stronger tendency to inhibit emotional expression, which may contribute to cumulative psychological strain over time [4]. Social support, defined as assistance provided to individuals in challenging situations, can reduce anxiety and aid adaptation, particularly in challenging circumstances such as hospitalization due to threatened preterm labor. Specifically, higher levels of perceived support have been linked to lower reliance on emotional suppression and greater emotional openness, both of which are associated with better psychological outcomes [5]. In high-stress contexts such as hospitalization for threatened preterm labor, social support may buffer emotional burden and influence the individual’s choice of emotion regulation strategies, including the extent to which negative emotions are suppressed [6].

Preterm birth is defined as the delivery of a live infant before the completion of 37 weeks of gestation, i.e., before day 259 from the first day of the last menstrual period. It presents a major clinical challenge for obstetricians and neonatologists, as it remains one of the leading causes of neonatal morbidity and mortality worldwide [7]. According to the 2024 World Health Organization (WHO) report, over 13 million babies are born prematurely each year—accounting for more than 10% of all live births—and this prevalence continues to rise [8]. The etiology of preterm birth is complex, involving both medical factors, such as infections or cervical insufficiency, and psychosocial factors, including anxiety, stress, poor social support, and socioeconomic adversity. Stress and anxiety are significant predictors of pregnancy complications, as they can affect placental function and hormonal processes that regulate the course of pregnancy, potentially leading to premature delivery [9].

The chronic suppression of emotions may lead to an accumulation of emotional tension, increasing the risk of depression, anxiety disorders, panic attacks, and cognitive impairments. Additionally, emotional suppression may contribute to psychosomatic conditions such as tension headaches and muscle pain, digestive issues (e.g., irritable bowel syndrome—IBS), sleep disorders (e.g., insomnia, frequent awakenings), hypertension, cardiac arrhythmias, and dermatological conditions (e.g., eczema, psoriasis). Emotional suppression can dysregulate the endocrine system, potentially impairing fertility [10].

Given these risks, social support emerges as a crucial protective factor in the relationship between anxiety and emotional suppression in health threats like preterm birth. Research indicates that emotional support not only reduces anxiety levels but also enhances emotion regulation capacity by lowering stress hormone levels such as cortisol [5]. Wang et al. showed that both social support and psychological resilience mediate the relationship between self-efficacy and prenatal stress levels [11]. This suggests that women who receive more support are better able to cope with stress and demonstrate a greater capacity for emotional regulation. In the context of women hospitalized due to threatened preterm labor, social support may reduce anxiety while also fostering emotional adaptation during this challenging health situation [12]. The above patterns can be explained by various dimensions of perceived social support, namely experienced assistance in cognitive, emotional, and instrumental domains. These dimensions serve as resource gains, enhancing psychological resilience in stressful life situations, including stress associated with perceived threats to life or health among women facing threatened preterm birth. The functional dimensions of perceived social support in such situations are linked to the motivation to acquire essential cognitive resources (which help in interpreting the reality of the situation), emotional resources (aimed at reducing negative emotions or enhancing positive ones), and/or instrumental resources (providing guidance for action under the circumstances). These forms of support help protect against stress resulting from real or anticipated resource loss, especially concerning the perceived threats to life and/or health of the unborn child [13,14].

Accordingly, this study aims to investigate the associations between anxiety levels, social support, and emotional suppression in women hospitalized due to threatened preterm labor, with particular emphasis on whether social support mediates the relationship between pregnancy-related anxiety and emotional suppression.

## 2. Materials and Methods

### 2.1. Study Design and Participants

This study employed a quantitative, observational design and was followed the STROBE (Strengthening the Reporting of Observational Studies in Epidemiology) guidelines to ensure methodological rigor. The mediation component of the study was reported in accordance with the AGReMA (A Guideline for Reporting Mediation Analyses) recommendations to ensure transparency regarding model specification, assumptions, and interpretation. Data collection took place in Poland between December 2024 and June 2025 and involved 213 pregnant women hospitalized due to threatened preterm labor at the Podkarpackie Voivodeship Hospital in Krosno (Poland).

**Figure 1:**
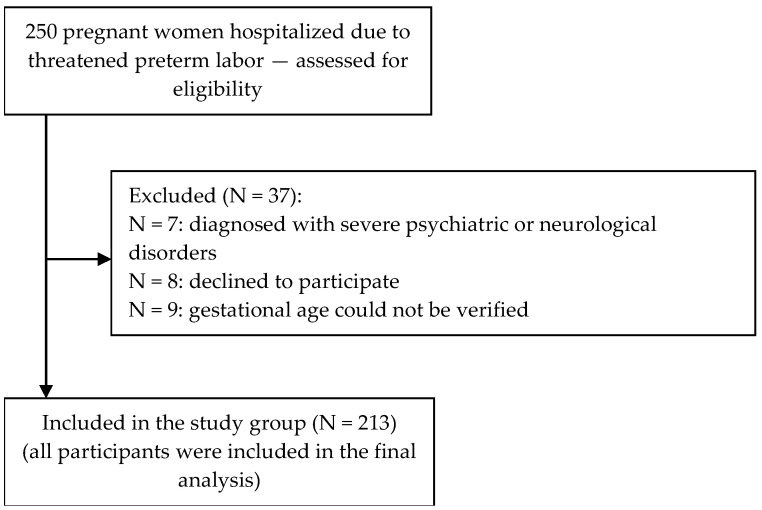
Flowchart of the recruitment process of the study participants.

The inclusion criteria were age ≥ 18 years, gestational age between 22 and 37 weeks, absence of diagnosed severe psychiatric or neurological disorders, and adequate psychophysical condition enabling informed completion of the study instruments. The exclusion criteria included age < 18 years, gestational age outside the 22–37-week range, diagnosis of severe psychiatric or neurological disorders, physical condition precluding participation, and inability to provide informed consent. Threatened preterm labor was diagnosed based on the presence of regular uterine contractions and/or cervical shortening or dilation occurring before 37 completed weeks of gestation. The diagnosis was made in accordance with national clinical guidelines and was consistent with the World Health Organization (WHO) definition of preterm labor. Gestational age was verified using medical documentation. Standard clinical management of threatened preterm labor at the participating hospital followed evidence-based national protocols. It included pharmacological interventions such as tocolytic therapy (e.g., atosiban) to inhibit uterine contractions and corticosteroid administration (e.g., intramuscular betamethasone) to accelerate fetal lung maturation. Patients were also prescribed bed rest, adequate hydration, and continuous monitoring of fetal well-being (cardiotocography) and uterine activity. Additional care included psychological support and patient education when needed. Questionnaires with missing responses on any item in the BSSS, STAI, or CECS were excluded listwise from analyses. The final analytic sample (*n* = 213) consisted only of participants with complete data on all study variables (Figure 1).

### 2.2. Instruments

The study was conducted using a diagnostic survey method with standardized questionnaires. The instruments included the Berlin Social Support Scales (BSSS), the State–Trait Anxiety Inventory (STAI), the Courtauld Emotional Control Scale (CECS), and a structured interview questionnaire designed to collect sociodemographic and clinical information about the participants.

The Berlin Social Support Scales (BSSS), developed by Schwarzer and adapted into Polish by Łuszczyńska and Kowalska, consist of six subscales designed to assess both cognitive and behavioral aspects of social support. For the purpose of this study, five subscales were used: Perceived Available Support (8 items), Need for Support (4 items), Support Seeking (5 items), Currently Received Support (15 items), and Buffering–Protective Support (6 items). The sixth subscale, Provided Support, was not included in the analysis. Additionally, within the Currently Received Support subscale, separate scores were calculated for emotional, instrumental, and informational support. Responses were rated on a four-point Likert scale ranging from 1 (strongly disagree) to 4 (strongly agree), with higher scores indicating greater levels of social support. The internal consistency of the BSSS subscales, as measured by Cronbach’s alpha coefficients in the study, was 0.78 for perceived social support, 0.83 for received social support, 0.81 for need for support, 0.72 for support seeking, and 0.78 for protective buffering [15].

The State–Trait Anxiety Inventory (STAI), developed by Spielberger, Gorsuch, and Lushene, consists of two separate 20-item subscales: STAI X-1, which measures state anxiety, and STAI X-2, which measures trait anxiety. In accordance with Polish norms, state anxiety scores ranging from 20 to 35 (sten scores 1–3) are considered low, scores from 36 to 52 (sten scores 4–7) indicate moderate anxiety, and scores from 55 to 80 (sten scores ≥8) reflect high anxiety levels. For trait anxiety, scores from 20 to 30 (sten scores 1–3) indicate low levels, 31 to 44 (sten scores 4–7) moderate levels, and 45 to 80 (sten scores ≥ 8) high levels of anxiety. The internal consistency of both subscales, as assessed by Cronbach’s alpha values were as follows: STAI X-1: α = 0.89, STAI X-2: α = 0.87 [16].

The Courtauld Emotional Control Scale (CECS) by Watson and Greer, adapted into Polish by Juczyński, includes three subscales measuring the suppression of anger, depression, and anxiety. Each subscale consists of seven items. The total emotional suppression score is calculated as the sum of all subscales and ranges from 21 to 84, with higher scores indicating greater suppression of negative emotions. Individual subscale scores range from 7 to 21 points. All analyses treated the total CECS score as an indicator of greater suppression of negative emotions, with higher scores reflecting stronger suppression. This interpretation was consistently applied across all statistical models and tables. The internal consistency coefficients (Cronbach’s α) reported for the Polish version were 0.80 for anger suppression, 0.77 for depression suppression, 0.78 for anxiety suppression, and 0.87 for the total emotional suppression index [17].

Anxiety was assessed using the STAI on the first day of hospitalization. Assessments of social support (BSSS) and emotional control (CECS) were conducted no earlier than 48 h after admission. This delay was based on the assumption that responses regarding emotional regulation and perceived social support would be more accurate and reliable following an initial adaptation period to hospital conditions, estimated at 48–72 h. The aim was to minimize the influence of acute stress on self-reported measures and to ensure that participants had experienced initial interactions with medical staff and, when possible, contact with family members—both of which can significantly shape perceptions of social support during hospitalization [18,19]. While we acknowledge that this temporal separation may limit causal inference, it was implemented to enhance ecological validity in a clinical setting. Given the cross-sectional design, all mediation results are interpreted as associative rather than causal.

### 2.3. Data Analysis

Statistical analyses were conducted using STATISTICA 13.0 software (StatSoft, Kraków, Poland). Descriptive statistics were presented for the examined variables. For continuous variables, the mean (M), standard deviation (SD), median (Me), minimum (Min), and maximum (Max) were reported. For categorical variables, frequencies (*n*) and percentages (%) were used. To examine relationships between selected variables, Pearson’s r correlation was used for continuous variables and Spearman’s rho for ordinal variables. The level of statistical significance was set at *p* < 0.05.

Mediation analysis was conducted using the medmod module in Jamovi 2.5.6 software, which implements GLM-based mediation models and corresponds to Model 4 of the PROCESS macro for simple mediation. Standardized coefficients were reported to examine the potential mediating role of social support. The analysis was based on a theoretically informed sequence of variables: anxiety (measured at hospital admission) was modeled as the independent variable (X), social support (measured 48–72 h later) as the mediator (M), and emotional suppression (also assessed after 48–72 h) as the dependent variable (Y). This ordering reflects a quasi-temporal structure designed to minimize acute stress effects on self-reports and approximate directional associations. A graphical representation of the hypothesized relationships is provided in the Supplement (Figure S1).

**Figure 2:**
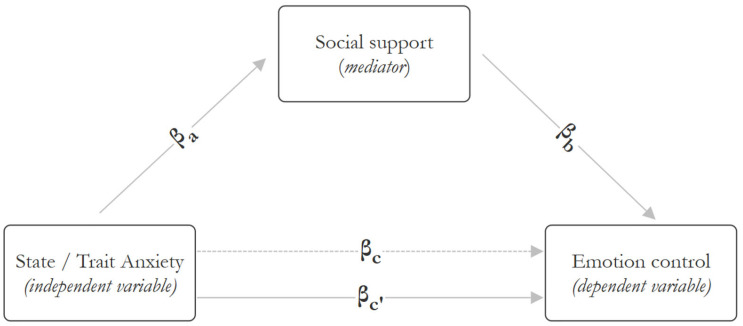
Relationships between variables in the mediation analysis.

In this study, the independent variable was the level of anxiety, assessed using the STAI X-1 (state anxiety) and STAI X-2 (trait anxiety) in separate models (Figure 2). The outcome variable, emotional suppression, was assessed using the Courtauld Emotional Control Scale (CECS), which measures the suppression of anger, anxiety, and depression in response to stress. The mediating variable was social support, measured using the Berlin Social Support Scales (BSSS). Prior to the analysis, the assumptions for mediation were verified: the independent variable was associated with the mediator; the mediator was associated with the dependent variable; and the direct effect of the independent variable on the dependent variable was attenuated after accounting for the mediator. The total effect (βc), indirect effect (βa × βb), and direct effect (βc′) were estimated. Percentile-based, two-tailed confidence intervals were calculated from 1000 bootstrap samples. Mediation was considered significant when the 95% CI for the indirect effect did not include zero [20].

This study included 213 women hospitalized due to threatened preterm labor in a single regional hospital. Given the relatively small total population of eligible patients during the study period, the sample can be considered a nearly complete population of interest. To assess statistical adequacy for mediation analysis, we performed a post hoc power estimation. For a medium-sized mediation effect (Cohen’s *f*^2^ = 0.15) and a significance level of α = 0.05, a minimum sample of approximately 100 participants is recommended to achieve 80% power [21]. Our final sample size of 213 participants provides sufficient statistical power (>0.80) to detect medium-sized indirect effects, and is therefore considered appropriate for the analytic strategy used in this study.

### 2.4. Ethical Considerations

The study was conducted in accordance with the principles of the Declaration of Helsinki and received a positive opinion from the Bioethics Committee at the State University of Applied Sciences in Krosno (approval number: 5/24). Approval for the study was also obtained from the head of the medical institution and the head of the hospital department.

To minimize psychological burden, only standardized, validated self-report instruments were used, assessing non-invasive psychological constructs. Before participation, all respondents received detailed information about the purpose of the study, the voluntary nature of their involvement, and the anonymity of data collection. Participants were explicitly informed that they could withdraw at any time without providing a reason, and that refusal to participate would not affect their medical care. In case any participant experienced distress during or after completing the questionnaires, on-site psychological support was available as part of standard perinatal care.

To ensure privacy and confidentiality, questionnaires were completed anonymously in isolated hospital rooms without the presence of medical staff. Data were stored in password-protected systems, in accordance with the European Union’s General Data Protection Regulation (GDPR) and institutional data protection policies.

No adverse psychological responses were reported during or after data collection.

## 3. Results

### 3.1. Characteristics of the Study Group

Table 1 presents the sociodemographic and obstetric characteristics of the study participants. The women were aged between 18 and 43 years, with a mean age of 29.64 ± 5.24 years. The majority lived in rural areas (68.54%), had higher education (50.70%), were married (90.61%), were employed (69.01%), and rated their socioeconomic conditions as good (70.42%). More than half were experiencing their first pregnancy (58.69%), and most reported that the pregnancy was planned (82.63%). The mean gestational age at the time of data collection was 30.41 ± 4.38 weeks.

### 3.2. Mean Scores of the Berlin Social Support Scales (BSSS), State–Trait Anxiety Inventory (STAI), and Courtauld Emotional Control Scale (CECS)

In the area of social support, the highest scores were recorded for Currently Received Support (54.20 ± 10.31) and Perceived Available Support (28.83 ± 4.77), while the lowest were observed for Need for Support (12.02 ± 1.71) and Buffering and Protective Support (12.29 ± 4.53). The mean score on the State Anxiety Inventory (STAI X-1) was 44.08 ± 10.59, and on the Trait Anxiety Inventory (STAI X-2) 39.75 ± 9.99. In terms of emotional suppression, the overall score on the Courtauld Emotional Control Scale (CECS) was 48.13 ± 13.04, with the highest value observed in the Anxiety Suppression subscale (16.80 ± 4.90) (Table 2).

### 3.3. Correlation Analysis

A correlation analysis was conducted to examine relationships between psychological variables, including trait anxiety, state anxiety, social support subscales, and emotional suppression (Table 3). Significant negative correlations were found between both types of anxiety and social support. Higher levels of Perceived Available Support and Currently Received Support were associated with lower levels of trait anxiety (r = −0.299, *p* < 0.05; r = −0.489, *p* < 0.05) and state anxiety (r = −0.459, *p* < 0.05; r = −0.548, *p* < 0.05). In contrast, Support Seeking and Buffering–Protective Support were positively correlated with both trait anxiety (r = 0.135, *p* < 0.05; r = 0.371, *p* < 0.05) and state anxiety (r = 0.211, *p* < 0.05; r = 0.494, *p* < 0.05).

The expression of negative emotions was also positively associated with anxiety levels. A stronger tendency to suppress symptoms of depression (r = 0.139, *p* < 0.05) and anxiety (r = 0.159, *p* < 0.05) was linked to higher state anxiety. Moreover, trait anxiety was significantly associated with a greater tendency to suppress negative emotions across all CECS subscales, with correlation coefficients ranging from r = 0.344 to r = 0.444.

### 3.4. Mediation Analysis

A mediation analysis was conducted to examine the role of social support as a mediator explaining the mechanism underlying the relationship between state anxiety (independent variable) and emotional suppression (dependent variable). Path analysis revealed that Perceived Available Support fully mediated the relationship between state anxiety and general emotional suppression (95% CI: 0.03961–0.2217; *p* = 0.001). This suggests that, after including the mediator, the direct effect of state anxiety on emotional suppression was no longer statistically significant. A trend toward mediation was observed for Need for Support, but the effect did not reach statistical significance (95% CI: −0.00482 to 0.0915; *p* = 0.076). In the component paths, higher state anxiety was significantly associated with lower perceived available support (β = –0.2991, *p* < 0.001), which in turn predicted lower emotional suppression (β = –0.3456, *p* < 0.001). In contrast, Buffering and Protective Support showed an opposite pattern: women with higher levels of state anxiety reported higher levels of buffering support (β = 0.3719, *p* < 0.001), which was associated with greater emotional suppression (β = 0.2727, *p* < 0.001). Mediation effects for Support Seeking and Currently Received Support were not statistically significant (*p* > 0.05) (see Table 4).

Table 5 presents the results of a mediation analysis examining the role of social support in the relationship between trait anxiety (independent variable) and emotional suppression (outcome variable). Perceived Available Support partially mediated this relationship (95% CI: 0.0691–0.3099; *p* < 0.001), indicating that the direct effect of trait anxiety on emotional suppression remained significant but was attenuated after including this mediator. Need for Support also played a significant mediating role, though the effect was weaker (95% CI: 0.0049–0.1250; *p* = 0.025). Buffering–Protective Support had a significant positive mediating effect—individuals with higher levels of trait anxiety reported higher levels of this support, which was associated with better emotional suppression (95% CI: 0.0379–0.2304; *p* = 0.007). An opposite pattern was observed for Currently Received Support—individuals with higher trait anxiety tended to receive less support, which negatively impacted emotion regulation (β = −0.1043; *p* = 0.018).

## 4. Discussion

Threatened preterm labor is a clinical condition that may trigger intense emotional responses in pregnant women, including anxiety, anger, guilt, and low mood [6,22]. Coping with these emotions and their implications for mental and physical health depends on psychosocial resilience resources, particularly emotional regulation abilities and the level of social support received [23,24]. The aim of the present study was to analyze the mediating role of social support in the relationship between anxiety and emotional suppression. The findings indicate that different forms of social support are differently associated with this process.

### 4.1. Pregnancy-Related Anxiety

State anxiety is characterized by high variability and intensity depending on the situation, which means it can intensify in stressful circumstances such as hospitalization due to threatened preterm labor [25]. In contrast, trait anxiety refers to a relatively stable disposition to respond with anxiety and reflects a tendency to interpret neutral or mildly stressful situations as threatening, which may hinder adaptation to hospitalization and increase the risk of emotional disturbances [26].

In this study, the STAI scale was used to measure anxiety, assessing it both as a transient emotional state and as a stable personality trait. The findings indicate that women hospitalized due to threatened preterm labor experienced moderate levels of both state and trait anxiety. Similar levels of anxiety have been reported in studies of pregnant women hospitalized for pregnancy complications in New Zealand and Turkey. Cornsweet Barber and Starkey compared anxiety levels in hospitalized pregnant women with those in a non-hospitalized control group. Their results showed that hospitalization was associated with significantly higher anxiety levels, with STAI scores reflecting a moderate level—consistent with the current study. The authors also highlighted that hospitalization, uncertainty about pregnancy outcomes, and lack of control can significantly heighten anxiety in pregnant women [27]. Yeşilçınar et al. likewise reported moderate levels of state anxiety, with key contributing factors including prior birth experiences, level of social support, and socioeconomic status [3]. These authors emphasized the importance of anxiety-reduction strategies such as psychological support and prenatal education, particularly among women hospitalized due to threatened preterm birth. Anxiety reflects an internal experience of actual or anticipated loss of resilience-related resources. In such situations, human activity is oriented toward conserving or increasing those resources. One such psychological resource is social support [13]. The current study found that support-seeking behavior significantly correlates with anxiety, suggesting that individuals with higher anxiety levels are more likely to seek support. Anxiety levels were negatively correlated with perceived available support and currently received support, indicating that individuals experiencing stronger anxiety perceived support as less accessible and were less likely to receive tangible help. On the other hand, Buffering–Protective Support was positively correlated with trait anxiety, suggesting that more anxious individuals may more actively utilize stress-buffering mechanisms of social support. Elevated state anxiety in women hospitalized due to threatened preterm labor represents a key psychological factor that may impact the course of pregnancy and perinatal outcomes [28]. The strong association found in this study between state anxiety and trait anxiety suggests that individuals who frequently experience anxiety in difficult situations also tend to exhibit higher levels of anxiety as a personality trait. Research by García-Blanco et al. showed that pregnant women with high anxiety levels reported not only increased subjective distress but also elevated stress biomarkers, such as cortisol, which may contribute to shortened gestational duration [29]. Moreover, pregnancy-related anxiety can impact hypothalamic–pituitary–adrenal (HPA) axis function, increasing the risk of uterine contractions and the onset of preterm labor [30]. A high level of anxiety during pregnancy is also a known risk factor for perinatal depression [31].

### 4.2. The Effects of Emotional Suppression

Chronic suppression of difficult emotions may have negative consequences for both mental and physical health. The overall results on the Courtauld Emotional Control Scale (CECS) indicated a moderate level of emotional suppression in the study group. The highest scores were observed in the subscale “anxiety suppression,” suggesting that the women more frequently used suppression strategies for anxiety than for other negative emotions. Hospitalization due to threatened preterm labor is an extremely stressful experience and can significantly impact the emotional state of pregnant women. The use of emotional suppression strategies—particularly anxiety suppression—may function as a defensive mechanism aimed at coping with intense stress and uncertainty related to the health of the fetus and the mother herself [27]. However, studies indicate that long-term emotional suppression, especially of anxiety, can be harmful and may deteriorate maternal mental health, potentially negatively affecting pregnancy outcomes and fetal development [30,32]. In the process of coping with negative emotions, as demonstrated in the current study, social support plays a crucial role. Individuals who perceive support as accessible and receive more support are better at emotional expression. Moreover, Buffering–Protective Support was positively correlated with emotion regulation, suggesting that individuals utilizing this form of support may be more inclined toward conscious emotional regulation. The ability to regulate emotions among women experiencing threatened preterm labor was evaluated in relation to anxiety levels, with elements of social support serving as mediators. All participants were hospitalized during data collection, which could have additionally affected their emotional state. Emotional control and anxiety are factors closely linked to concerns about pregnancy progression and fetal well-being, and also associated with pain perception during pregnancy and labor [33]. Anxiety activates the sympathetic nervous system, leading to the release of stress hormones (e.g., cortisol), which intensify labor pain, inhibit oxytocin production, and prolong labor duration.

Identifying ways to maximize comfort while minimizing the risk of complications is a key component of maternal care. Among the recommended non-pharmacological methods for reducing both pain and anxiety during labor are music therapy, aromatherapy, and relaxation techniques [34]. In a randomized controlled trial, Goetz et al. demonstrated the effectiveness of mindfulness-based interventions among women hospitalized for threatened preterm labor [35]. These techniques significantly reduced state anxiety and improved both well-being and emotional coping capacity. The ability to manage emotions—viewed as a component of emotional intelligence—along with the perception of available social support, serves as a protective factor against psychological stress. Emotional intelligence refers to the capacity to perceive, assess, and express emotions, as well as to understand their influence on thought processes and problem-solving. High emotional intelligence is associated with better mental health and lower levels of anxiety [36].

### 4.3. Social Support

Social support, both from medical staff and close relatives, can mitigate the effects of anxiety, increase the sense of safety, and improve emotional regulation in pregnant women. Importantly, its impact is amplified when the support is pregnancy-specific, encompassing not only emotional and practical help but also structured assistance within antenatal care systems [37]. Support from a partner and medical staff during hospitalization plays a unique role in modulating stress responses and facilitating emotional regulation in high-risk pregnancies [38]. Moreover, perceived support availability rather than objectively received support has been found to be a stronger predictor of psychological outcomes in pregnant women [39]. Additionally, the context of hospitalization itself may influence how support is perceived. Factors such as separation from familiar support networks, limited visiting hours, dependency on clinical staff, and the unfamiliar hospital environment can reduce the perceived availability of social support, even when objective support is present. An integrative review by Crane et al. highlighted that even non-traditional forms of support, such as art-based interventions, can provide meaningful emotional benefits during pregnancy. These interventions foster expression of complex emotions, build a sense of connection, and enhance personal resourcefulness key components of psychosocial resilience. As such, pregnancy-related social support should be conceptualized broadly, encompassing both informal interpersonal and professionally facilitated therapeutic or creative interventions [40].

Mediation analysis suggested that perceived available support statistically mediated the relationship between state anxiety and emotional suppression, suggesting that women with higher anxiety levels who perceived support as available exhibited better emotional regulation mechanisms and less suppression of negative emotions. These findings align with results reported by Bedaso et al., which showed that high levels of perceived social support can serve as a crucial buffer in stressful situations, reducing psychological distress associated with hospitalization and the risk of preterm birth [41]. Similarly, Zhou et al. emphasized the importance of perceived social support in modifying the effects of prenatal depression and anxiety, leading to improved pregnancy outcomes [42]. Consistent patterns have also been observed in other populations: studies from New Zealand [27], Turkey [3], and China [12] demonstrated that perceived social support significantly reduces anxiety levels and improves emotional well-being in pregnant women, regardless of cultural context.

Instrumental and emotional support function as protective buffers against challenges experienced during the transition to parenthood, safeguarding maternal mental health. Research indicates that women who perceive their environment as supportive—emotionally and practically—experience lower levels of anxiety and greater adaptive capacity in stressful situations such as pregnancy complications or hospitalization [43]. For pregnant women requiring hospitalization, it is therefore essential to integrate emotional support and stress-coping strategies into comprehensive high-risk pregnancy care. Kao et al. found that pregnant women hospitalized for threatened preterm labor who received greater support from partners and medical personnel reported lower levels of anxiety and better adaptation to hospitalization [44]. Social support in such cases may act in several ways:

1. By direct emotional impact, reducing anxiety and stress levels [45];
2. By increasing access to coping strategies and mobilizing women’s psychological resources to adapt and face the challenges of pregnancy, including developing maternal identity [46,47].

Importantly, not only the availability of support matters, but also how it is perceived by the patient. Studies indicate that women who subjectively perceive support as insufficient, even if help is available, may not benefit from its protective effects on mental health. Therefore, the delivery of support, its communication, and personalization to meet women’s individual needs are crucial [48].

In this study, Currently Received Support did not mediate the relationship between anxiety and emotional suppression. However, a significant negative correlation was found—women who received less support during hospitalization experienced higher anxiety levels. Support during the perinatal period is influenced by relationship status and relationship quality; support from a partner or mother appears more valuable than support from friends or extended family. Interestingly, some evidence suggests that being a single mother may be less detrimental than being in an unsupportive relationship. Women who perceived their partners as unsupportive or ambivalent were more likely to experience perinatal depression and reported a greater need for support from alternative sources [42,49].

The role of social support in pregnancy and childbirth was particularly emphasized during the COVID-19 pandemic, when infection control measures led to restrictions on support persons during labor. The absence of close relatives during childbirth was associated with increased stress and anxiety. Furthermore, virtual support did not effectively alleviate heightened emotional distress. These findings highlight the irreplaceable value of in-person support during labor and delivery. Beyond mere presence, the quality of engagement and attentiveness of the support person is essential [13,50].

In conclusion, our findings support a significant statistical mediating role of perceived available social support in the relationship between anxiety and emotion regulation among pregnant women hospitalized due to threatened preterm labor. The results suggest that higher perceived support may be linked to greater emotional regulation and less suppression of negative affect, underscoring the need for strategies that enhance the subjective sense of support. This includes educational programs, psychological interventions, and the involvement of healthcare professionals and family members, which may contribute to reduced anxiety in this vulnerable population [49].

The positive mediating effect of Buffering–Protective Support in the relationship between high trait anxiety and improved emotional regulation among women at high risk of preterm birth reflects an important resilience mechanism. In the face of intense stress (caused by real or anticipated resource loss), individuals tend to attribute greater significance to perceived resource gains (i.e., social support) to halt the spiral of loss, balance deficits, and/or expand their coping resources, which may be associated with improved emotional self-regulation [13].

### 4.4. Strengths and Limitations

The inclusion of mediation analysis allowed for a more precise understanding of the mechanisms underlying the relationship between anxiety and emotional suppression, with particular emphasis on the role of social support. The findings provide valuable insights into the emotional regulation processes among hospitalized women at risk of preterm labor, which may have practical implications for perinatal care and the design of psychological interventions. All standardized instruments had been culturally adapted and validated for the Polish population, supporting their appropriateness in this context.

However, the study was conducted in a single hospital in southeastern Poland, which may limit generalizability to other regions or healthcare systems. Limiting the sample to hospitalized patients may further constrain external validity, particularly with regard to women managed on an outpatient basis. Moreover, the sample was relatively demographically homogeneous, with all participants recruited from a culturally and ethnically uniform population in a specific geographic area. This homogeneity, while useful for controlling contextual factors, may limit the applicability of the findings to more diverse populations or healthcare settings. Future studies should compare inpatient and outpatient populations to better understand context-dependent differences in anxiety, emotion regulation, and perceived social support.

Given the cross-sectional and observational design, causal relationships cannot be definitively established. While mediation analysis provides insight into possible mechanisms, the directionality of effects remains tentative. Traditional mediation techniques, rather than formal causal inference models, were used; thus, findings should be interpreted as statistical associations rather than causal pathways. Future research using counterfactual or longitudinal designs is warranted.

Alternative explanations should also be considered. For example, women with better emotion regulation may perceive support more positively, or unmeasured factors (e.g., personality traits, prior mental health status) may influence both anxiety and perceived support. Furthermore, reverse causation cannot be ruled out; it is plausible that higher levels of emotional suppression could intensify perceived anxiety, rather than the other way around. Although the temporal ordering of variables was theoretically informed and partially separated in time, the cross-sectional nature of the design prevents firm conclusions about the directionality of effects. These possibilities further emphasize the need for cautious interpretation.

Another limitation is the temporal separation of measurements. We assumed that allowing 48–72 h for initial adaptation to hospital conditions would yield more reliable and stable self-reports, particularly in a high-stress environment such as emergency obstetric care. This decision was based on existing literature suggesting that acute anxiety responses to hospitalization typically stabilize within this window. However, it may also introduce unmeasured confounding influences over time and limits the ability to draw causal conclusions from the mediation analysis.

Compared to the postpartum period, the mental health of pregnant women receives less clinical and research attention. There is a clear need for comprehensive studies on risk factors for pregnancy-related anxiety as well as protective mechanisms that support emotional regulation during this critical period.

A better understanding of these relationships could contribute to the development of effective screening strategies to identify women at increased risk of elevated anxiety during pregnancy. Further research is also needed to explore women’s life experiences and their subjective perception of social support during pregnancy. In particular, longitudinal designs following women from hospital admission to the postpartum period could examine whether changes in perceived support predict improvements in anxiety and emotion regulation. Such prospective studies would allow for stronger inferences about causality and inform the timing and content of psychosocial interventions [2,43].

### 4.5. Implications for Policy and Practice

Anxiety during pregnancy may intensify in the third trimester, particularly in complicated pregnancies requiring hospitalization. Women facing threatened preterm labor may be especially vulnerable to stress related to limitations imposed by withdrawal from professional and social activities. Special attention should be given to women whose pregnancy complications lead to early withdrawal from their everyday roles. The monotony of staying at home or in hospital, combined with a lack of social stimulation or inability to continue paid employment, may contribute to the development of mental health disorders, often described as a “sense of entrapment” [51,52]. The results of this study suggest that interventions aimed at strengthening social support may be an effective strategy for reducing the negative effects of anxiety in women at risk of preterm labor. Critical components include ensuring access to emotional and instrumental support from both family members and medical staff. Care protocols should also incorporate psychological mechanisms for emotion regulation and offer structured educational and psychotherapeutic interventions that help women adapt to complex health challenges.

While the statistical associations identified in this study were significant, the practical implications for clinical care are also noteworthy. Given that suppression may mask emotional dysregulation and reduce help-seeking behaviors, even modest reductions in suppression—such as those associated with greater perceived support in our study—may lead to clinically meaningful improvements in patients’ emotional functioning. These improvements may in turn facilitate better adaptation to hospitalization, improve patient–provider communication, and support maternal–infant bonding after birth.

Many of the recommended interventions, such as brief psychosocial screening tools, partner involvement, and basic emotional support from medical staff are low-cost and feasible to implement within standard hospital protocols, without requiring significant additional resources.

Screening for anxiety and perceived support can be conducted using short, validated tools by trained personnel during routine admission procedures. Interventions such as empathetic communication or encouragement of family presence can be implemented without major structural changes. More structured interventions, such as antenatal psychoeducation or mindfulness-based stress reduction, may require additional training or program integration but can be selectively offered to women with high trait anxiety or low perceived support. This tiered approach ensures efficient allocation of resources while addressing the psychological needs of women hospitalized with threatened preterm labor. Incorporating these elements into prenatal care may contribute to a more comprehensive approach to maternal mental health. Routine assessment of pregnant women’s psychosocial resources—including the availability of support, quality of family relationships, and presence of life stressors—should become standard practice in perinatal care.

## 5. Conclusions

Women hospitalized due to threatened preterm labor demonstrated moderate levels of both state and trait anxiety, indicating heightened psychological vulnerability in this patient group. State anxiety—amplified by hospitalization, uncertainty about pregnancy outcomes, and potential fetal complications—represents a significant and modifiable risk factor for emotional distress. Trait anxiety, as a stable predisposition, may further compromise emotional functioning and pregnancy adaptation over time.

Mediation analysis revealed that the relationship between anxiety and emotion regulation is not uniform and depends on the type of social support involved. Specifically, perceived available support fully mediated the relationship between anxiety and emotional control, suggesting that women who believe support is available are better equipped to manage negative emotions. In contrast, Buffering–Protective Support demonstrated an activating role, enhancing emotional control in women with elevated anxiety, rather than simply buffering its negative effects.

These findings underscore the need for targeted psychosocial assessments and interventions in obstetric care. Routine screening of perceived support and emotional regulation should be incorporated into prenatal care protocols for women at risk of preterm labor. Psychosocial interventions—such as partner-involved care, staff-delivered emotional support, and resilience-based education—should be initiated early during hospitalization to strengthen coping mechanisms.

Medical staff should recognize that not all forms of social support function uniformly. Effective perinatal care requires an individualized approach that considers both the subjective perception of support and the patient’s psychological profile. Enhancing perceived social support may function both as a protective factor and a therapeutic mechanism to improve emotional control, reduce anxiety, and potentially influence pregnancy outcomes.

## Figures and Tables

**Table 1:** Characteristics of the study group.

Characteristics of the Study Group		N	%
Mean age/years	M (SD) Me	29.64 (5.24) 30.00	
	Min–Max	18.00–43.00	
Residence	Urban	67	31.46
	Rural	146	68.54
Education	Primary	17	7.98
	Secondary	87	40.85
	Higher	108	50.70
Relationship status	Married	193	90.61
	Single	20	9.39
Professional status	Employed	147	69.01
	Unemployed	66	30.99
Financial situation	Very good	31	14.55
	Good	150	70.42
	Average	32	15.02
Number of pregnancies	1	125	58.69
	2	36	16.90
	≥3	52	24.41
Planned pregnancy	Yes	176	82.63
	No	37	17.37
Week of pregnancy	M (SD) Me	30.41 (4.38) 31.00	
	Min–Max	22.00–37.00	

**Table 2:** Mean scores on the Berlin Social Support Scales (BSSS), State–Trait Anxiety Inventory (STAI), and Courtauld Emotional Control Scale (CECS).

	Scale	M	Me	SD	Min	Max
Berlin Social Support Scales (BSSS)	Perceived Available Support	28.83	30.00	4.77	12.00	35.00
	Need for Support	12.02	12.00	1.71	6.00	16.00
	Seeking Support	14.82	15.00	2.99	7.00	20.00
	Currently Received Support	54.20	58.00	10.31	15.00	60.00
	Buffering and Protective Support	12.29	11.00	4.53	6.00	24.00
State–Trait Anxiety Inventory (STAI)	State Anxiety Scale STAI X-1	44.08	42.00	10.59	22.00	70.00
	Trait Anxiety Scale STAI X-2	39.75	39.00	9.99	20.00	70.00
Courtauld Emotional Control Scale (CECS)	Anger	15.50	16.00	4.64	7.00	27.00
	Depression	15.97	15.00	5.28	7.00	27.00
	Anxiety	16.80	17.00	4.90	7.00	28.00
	Total	48.13	47.00	13.04	21.00	80.00

**Table 3:** Correlations between anxiety levels, Berlin Social Support Scales (BSSS), and Courtauld Emotional Control Scale (CECS).

		STAI X1	STAI X2	Perceived Available Support	Need for Support	Seeking Support	Currently Received Support	Buffering and Protective Support
State–Trait Anxiety Inventory (STAI)	STAI X1	-	0.636 *	−0.299 *	0.135 *	−0.053	−0.489 *	0.371 *
	STAI X2	0.636 *	-	−0.459 *	0.211 *	0.005	−0.548 *	0.494 *
Berlin Social Support Scales (BSSS)	Perceived available support	−0.299 *	−0.459 *	-	0.044	0.267 *	0.609 *	−0.442 *
	Need for support	0.135 *	0.211 *	0.044	-	0.372 *	−0.129	0.113
	Seeking support	−0.053	0.005	0.267 *	0.372 *	-	0.048	0.114
	Currently received support	−0.489 *	−0.548 *	0.609 *	−0.129	0.048	-	−0.517 *
	Buffering and protective support	0.371 *	0.494 *	−0.442 *	0.113	0.114	−0.517 *	-
Courtauld Emotional Control Scale (CECS)	Total	0.168 *	0.444 *	−0.455 *	0.143 *	−0.223 *	−0.282 *	0.360 *
	Anger	0.056	0.344 *	−0.367 *	0.184 *	−0.130	−0.230 *	0.199 *
	Depression	0.139 *	0.398 *	−0.426 *	0.101	−0.214 *	−0.245 *	0.397 *
	Anxiety	0.159 *	0.351 *	−0.379 *	0.131	−0.149 *	−0.255 *	0.328 *

* *p* < 0.05.

**Table 4:** Mediation analysis results: the role of different forms of social support in the relationship between state anxiety and emotional suppression.

Type	Effect	Estimate	SE	95% CI LL	95% CI UL	β	*p*
Indirect	STAI X1 ⇒ PAS ⇒ CECS	0.1273	0.0399	0.0396	0.2217	0.1034	0.001
	STAI X1 ⇒ NFS ⇒ CECS	0.0403	0.0227	−0.0048	0.0915	0.0327	0.076
	STAI X1 ⇒ SS ⇒ CECS	0.0170	0.0222	−0.0167	0.0584	0.0138	0.445
	STAI X1 ⇒ CRS ⇒ CECS	−0.0575	0.0499	−0.1888	0.0728	−0.0467	0.249
	STAI X1 ⇒ BPS ⇒ CECS	0.1249	0.0387	0.0577	0.2184	0.1014	0.001
Component	STAI X1 ⇒ PAS	−0.1347	0.0295	−0.2031	−0.0557	−0.2991	<0.001
	PASt ⇒ CECS	−0.9445	0.2121	−1.4307	−0.4826	−0.3456	<0.001
	STAI X1 ⇒ NFS	0.0219	0.0110	−0.0025	0.0461	0.1354	0.046
	NFS ⇒ CECS	1.8413	0.4745	0.5979	3.0722	0.2417	<0.001
	STAI X1 ⇒ SS	−0.0151	0.0193	−0.0445	0.0148	−0.0533	0.436
	SS ⇒ CECS	−1.1272	0.2860	−1.7911	−0.4513	−0.2586	<0.001
	STAI X1 ⇒ CRS	−0.4766	0.0581	−0.6687	−0.2739	−0.4898	<0.001
	CRS ⇒ CECS	0.1207	0.1036	−0.1639	0.3622	0.0954	0.244
	STAI X1 ⇒ BPS	0.1591	0.0272	0.1059	0.2122	0.3719	<0.001
	BPS ⇒ CECS	0.7849	0.2028	0.3916	1.2130	0.2727	<0.001
Direct	STAI X1 ⇒ CECS	−0.0444	0.0818	−0.2123	0.1312	−0.0361	0.587
Total	STAI X1 ⇒ CECS	0.2074	0.0833	−0.0267	0.4163	0.1685	0.013

CI—Confidence interval; LL—Lower limit; UL—Upper limit; PAS = Perceived Available Support; NFS = Need for Support; SS = Seeking Support; CRS = Currently Received Support; BPS = Buffering and Protective Support; CECS = Emotional Suppression; STAI X1 = State Anxiety.

**Table 5:** Mediation analysis results: the role of different forms of social support in the relationship between trait anxiety and emotional suppression.

Type	Effect	Estimate	SE	95% CI LL	95% CI UL	β	*p*
Indirect	STAI X2 ⇒ PAS ⇒ CECS	0.18062	0.0515	0.0691	0.3099	0.1383	<0.001
	STAI X2 ⇒ NFS ⇒ CECS	0.05358	0.0239	0.0049	0.1250	0.0410	0.025
	STAI X2 ⇒ SS ⇒ CECS	−0.00189	0.0222	−0.0398	0.0440	0.0014	0.932
	STAI X2 ⇒ CRS ⇒ CECS	−0.13619	0.0573	−0.2756	0.0159	−0.1043	0.018
	STAI X2 ⇒ BPS ⇒ CECS	0.12734	0.00474	0.0379	0.2304	0.0975	0.007
Component	STAI X2 ⇒ PAS	−0.21943	0.0291	−0.2873	−0.1366	−0.4592	<0.001
	PAS ⇒ CECS	−0.82312	0.2076	−1.3004	−0.3817	−0.3012	<0.001
	STAI X2 ⇒ NFS	0.03620	0.0115	0.0067	0.0575	0.2112	0.002
	NFS ⇒ CECS	1.48002	0.4646	0.4443	2.5568	0.1942	0.001
	STAI X2 ⇒ SS	0.00175	0.0205	−0.0343	0.041	0.0058	0.932
	SS ⇒ CECS	−1.08290	0.2752	−1.6675	−0.4835	−0.2484	<0.001
	STAI X2 ⇒ CRS	−0.56633	0.0591	−07423	−0.3417	−0.5487	<0.001
	CRS ⇒ CECS	0.24048	0.0981	−0.0329	0.4653	0.1900	0.014
	STAI X2 ⇒ BPS	0.22430	0.0270	0.1835	0.2611	0.4944	<0.001
	BPS ⇒ CECS	0.56769	0.1998	0.1659	1.0072	0.1972	0.004
Direct	STAI X2 ⇒ CECS	0.35635	0.0922	0.1418	0.5685	0.2729	<0.001
Total	STAI X2 ⇒ CECS	0.57979	0.0803	0.3865	0.7484	0.4440	<0.001

CI—Confidence interval; LL—Lower limit; UL—Upper limit; PAS = Perceived Available Support; NFS = Need for Support; SS = Seeking Support; CRS = Currently Received Support; BPS = Buffering and Protective Support; CECS = Emotional Control; STAI X2 = Trait Anxiety.

## Data Availability

The data that support the findings of this study are available from the corresponding author upon reasonable request.

## Supplementary Materials

The following supporting information can be downloaded at https://www.mdpi.com/article/10.3390/jcm14176002/s1, Figure S1: Conceptual Directed Acyclic Graph illustrating assumed causal pathways between pregnancy anxiety, perceived social support, and emotional suppression.

## Abbreviations

The following abbreviations are used in this manuscript:

BSSS	Berlin Social Support Scales
CECS	Courtauld Emotional Control Scale
STAI	State–Trait Anxiety Inventory
STROBE	Strengthening the Reporting of Observational Studies in Epidemiology
WHO	World Health Organization
