# Supplementary material for: The Mediating Role of Social Support Between Pregnancy Anxiety and Emotional Suppression in Women with Threatened Preterm Labor
Source: J Clin Med. 2025 Aug 25;14(17):6002. doi: 10.3390/jcm14176002 (PMC12429850; doi:10.3390/jcm14176002)
Supplement: Supplementary file 1 [file jcm-14-06002-s001.zip › jcm-3806337-supplementary.pdf]

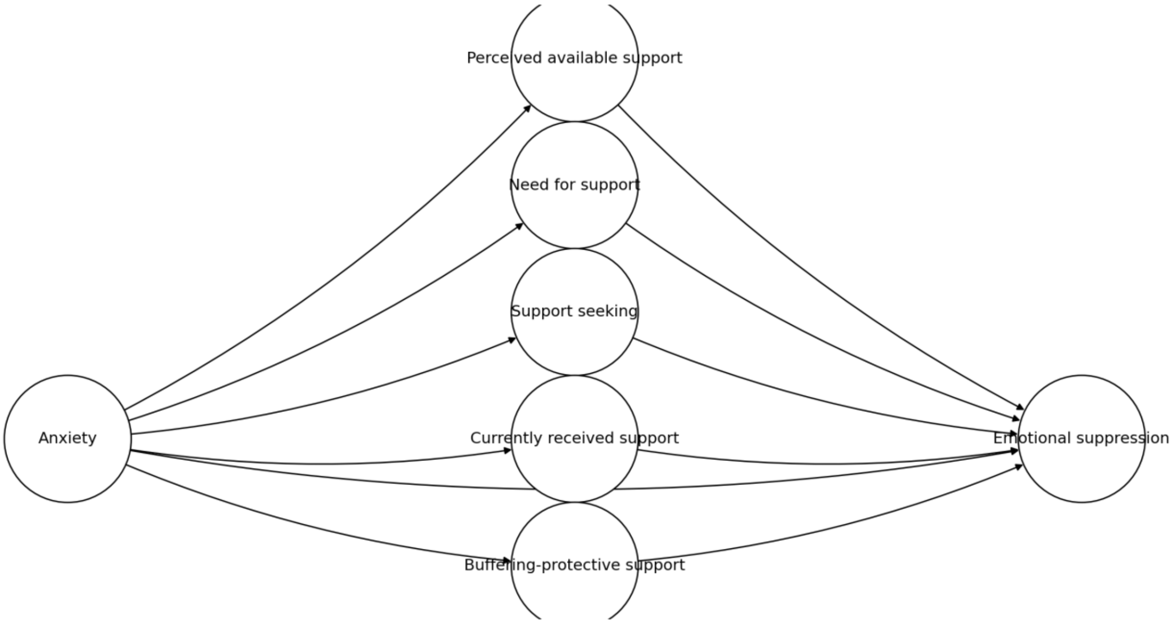

Figure S1. Conceptual Directed Acyclic Graph illustrating assumed causal pathways between pregnancy anxiety, perceived social support, and emotional suppression.
